# Effects of robot-assisted hand function therapy on brain functional mechanisms: a synchronized study using fNIRS and sEMG

**DOI:** 10.3389/fmed.2024.1411616

**Published:** 2024-10-31

**Authors:** Changfeng Cheng, Tiantian Liu, Beibei Zhang, Xubo Wu, Zhenwang Song, Zhongzhi Zhao, Xue Ren, Minjun Zhao, Yajuan Su, Jiening Wang

**Affiliations:** ^1^Shanghai Seventh People’s Hospital Affiliated to Shanghai University of Traditional Chinese Medicine, Shanghai, China; ^2^The first affiliated hospital of Anhui University of Traditional Chinese Medicine, Anhui, China

**Keywords:** robot-assisted hand function therapy, functional near-infrared spectroscopy, surface electromyography, brain function, stroke

## Abstract

**Background:**

Robot-assisted hand function therapy is pivotal in the rehabilitation of patients with stroke; however, its therapeutic mechanism remains elusive. Currently, research examining the impact of robot-assisted hand function therapy on brain function in patients with stroke is scarce, and there is a lack of studies investigating the correlation between muscle activity and alterations in brain function.

**Objective:**

This study aimed to investigate the correlation between forearm muscle movement and brain functional activation by employing the synchronized use of functional near-infrared spectroscopy and surface electromyography methods. Moreover, it sought to compare neural activity patterns during different rehabilitation tasks and refine the mechanism of robot-assisted hand function therapy for post-stroke hand function impairments.

**Methods:**

Stroke patients with hand dysfunction underwent three sessions of robot-assisted hand function therapy within 2 weeks to 3 months of onset. The fNIRS-sEMG synchronous technique was used to observe brain function and forearm muscle activation. Ten participants were randomly assigned to receive mirror, resistance, or passive rehabilitation training. During the intervention, cortical and muscle activation information was obtained using fNIRS and electromyographic signals. The primary outcomes included changes in oxyhemoglobin concentration and root mean square of surface electromyography.

**Results:**

Compared to the resting state, the Oxy-Hb concentration in the brain regions involved in three rehabilitation tasks with robot-assisted hand function therapy significantly increased (*p* < 0.05). Mirror therapy significantly enhanced the prefrontal cortex and the superior frontal cortex activation levels. In contrast, resistance therapy significantly promoted the activation of the supplementary motor area and the premotor cortex. Passive rehabilitation tasks showed some activation in the target brain area premotor cortex region. Robot-assisted hand function therapy has shown that forearm muscle movement is closely related to oxygenated hemoglobin concentration activity in specific brain regions during different rehabilitation tasks.

**Conclusion:**

The simultaneous sEMG-fNIRS study found a significant correlation between muscle movement and brain activity after stroke, which provides an important basis for understanding the treatment mechanism of hand function impairment.

## Introduction

Stroke, a prevalent neurological disease (1), is witnessing a global increase in incidence and disability rates (2). Among its complications, hand dysfunction stands out, with 38% of patients experiencing significant impairment after 3 months and approximately 30 to 66% failing to recover completely after 6 months (3, 4). This severe hand dysfunction significantly affects patients’ quality of life and their ability to perform daily activities (5).

Robot-assisted hand function therapy is crucial in restoring hand function post-stroke (6, 7). This therapy allows for personalized treatment plans based on individual patient needs, offering enhanced precision and control (8). Robot-assisted hand function therapy has effectively improved hand function in patients with impaired hand function (9).

Although robot-assisted therapy has been widely studied and applied in clinical Settings in the past few decades (10, 11), there are relatively few studies on the mechanism of robot-assisted hand function therapy on brain function and arm muscle activity (12). In this study, near infrared spectroscopy (fNIRS) and surface electromyography (sEMG) were used to investigate the potential mechanism of robot-assisted hand function therapy on brain function changes. The main objective was to study the correlation between brain function and muscle activity in patients with hand dysfunction. Secondly, the effect of robot-assisted hand function therapy on the activation level of cerebral cortex and the activation degree of arm muscle was studied. This study provides real-time and sensitive neural evaluation indicators for robot-assisted hand function treatment, which helps to better understand the differences in cortical activation under different rehabilitation modes, and formulate more reasonable and effective strategies for personalized rehabilitation of hand dysfunction.

## Methods

### Design overview

#### Study design

**Figure 1:**
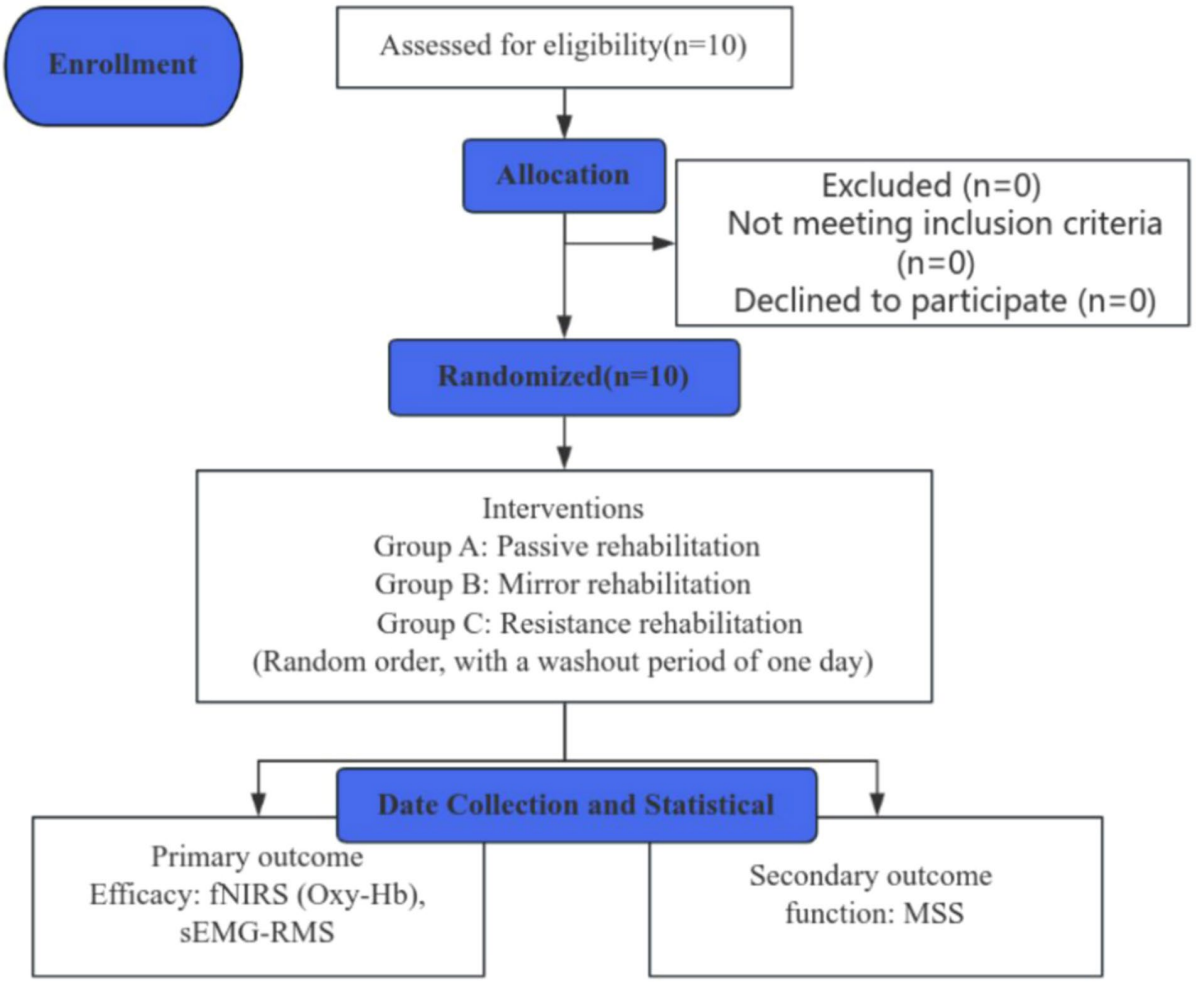
A concise flowchart depicting the entire study process. fNIRS, near-infrared spectroscopy; sEMG, surface electromyography; MSS, Motor Status Scale; RMS, root mean square; Oxy-Hb, oxyhemoglobin.

This study was a single-blinded, randomized, controlled clinical trial with a 3-day intervention and a 1-day washout period. Ten patients diagnosed with post-stroke hand dysfunction, recruited from the Rehabilitation Center of Shanghai Seventh People’s Hospital, were randomly allocated into Group A (mirror image rehabilitation), Group B (resistance rehabilitation), and Group C (passive rehabilitation). A schematic representation of the whole study is depicted in Figure 1. The study protocol was approved by the Ethics Committee of Shanghai Seventh People’s Hospital (2023-7th-HIRB-028) and registered by the Chinese Clinical Trial Center ChiCTR 2200063150.

### Participants

Participants who met the diagnostic criteria for stroke-related hand dysfunction outlined by the American Heart Association/American Stroke Association (13) were chosen. Further screening was performed against the inclusion and exclusion criteria.

#### Inclusion criteria

The inclusion criteria were as follows: (1) confirmed first onset of cerebrovascular disease through brain computed tomographic (CT) or magnetic resonance imaging (MRI) examination; (2) within 2 weeks to 3 months of onset; (3) age between 18 and 75 years; (4) education level of junior high school or higher; (5) Brunnstrom stage hand function above grade 2, indicating unilateral hand motor dysfunction; (6) absence of cognitive impairment, with a mini-mental state examination (MMSE) score of 25 or above; (7) willingness to participate and cooperate with treatment and examination; (8) signed informed consent provided by patients and their families.

#### Exclusion criteria

The exclusion criteria were as follows: (1) presence of infected or broken skin on the head; (2) poorly controlled epilepsy; (3) severe speech, attention, hearing, visual, intellectual, or mental disorders; cognitive impairment (as assessed by a simple cognitive status checklist: <25 points); (4) participation in other concurrent clinical trials; (5) inability to fully engage in training and evaluation for any reason.

#### Randomization and masking

Three random numbers were generated using the SPSS 26.0 software and placed into sealed, opaque envelopes. Participants were informed in advance that the training order would be randomized, and they would undergo three kinds of training sequentially, with no option to change the order. Participants meeting the inclusion criteria were randomly selected in the inclusion order and allocated to a group following the label instructions in the envelope.

Due to the particularity of robot-assisted hand function treatment, it is difficult to implement the double-blind method in this study (14), so we adopted the single-blind method. The evaluators were unaware of the grouping assignment of patients and the type of intervention they received.

### Interventions

#### Health education

All patients received comprehensive health education according to the guidelines provided by the American Heart Association/American Stroke Association (15). This accomplishment was facilitated by distributing informative brochures on stroke-related topics to enhance patients’ understanding. The brochures covered various issues, including stroke awareness, risk factors, rehabilitation options, psychological support, and dietary recommendations.

#### Robot-assisted hand function therapy

**Figure 2:**
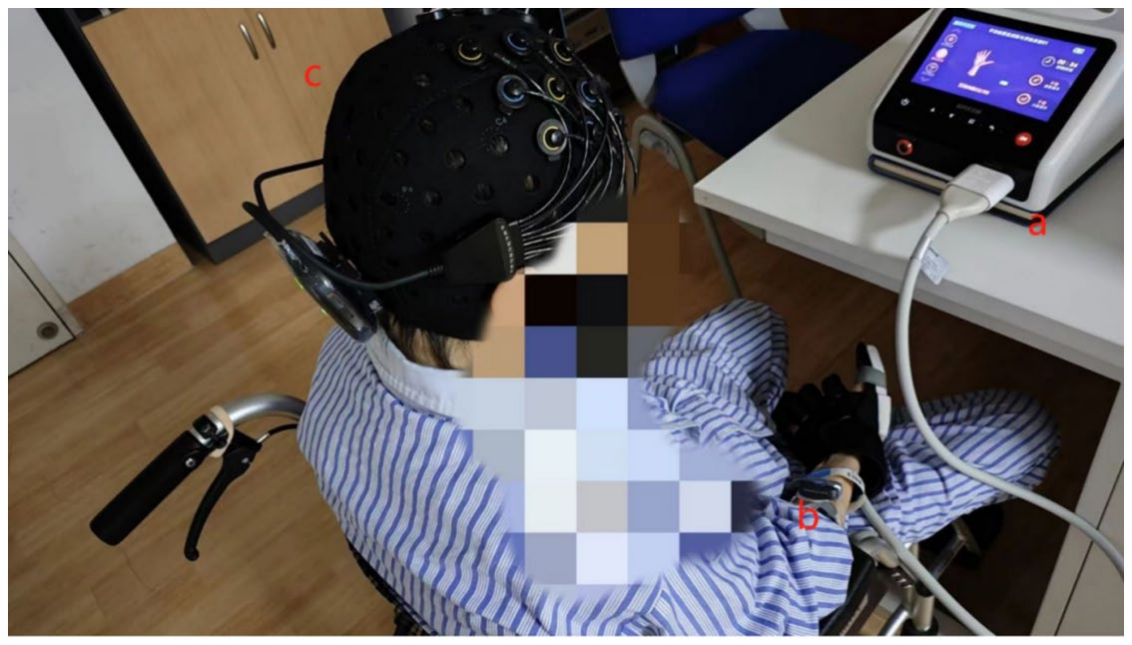
Training task for robot-assisted hand function therapy and data acquisition using fNIRS and sEMG. (a) Patients with robot-assisted hand function therapy gloves. (b) Electrodes placed on the forearm muscles. (c) Participants wearing a near-infrared test cap.

The bionic soft hand rehabilitation robot glove (Hunan Sirrem Medical Technology Co., Ltd.) was utilized for robot-assisted hand function therapy. Each rehabilitation session lasted 10 min, with consistent training intensity (duration and strength) for all patients. Ten patients, wearing gloves, sat in a quiet environment. Gloves were worn for all three treatments. The therapy comprises three distinct modalities (Figure 2):

##### Passive rehabilitation training group (Group A)

This mode involves the soft hand rehabilitation robot guiding the affected hand through grasping and finger movements. The finger movements are driven entirely by the robot, defining this modality as passive rehabilitation training.

##### Mirror rehabilitation training group (Group B)

Patients in this group observe their unaffected hand performing mirror-image grasping and finger movements displayed on the screen. Subsequently, they imagine their affected hand executing the same movements, aiming to synchronize both sides as closely as possible.

##### Resistance rehabilitation training group (Group C)

Patients observe finger movements on a screen and replicate them with their affected hand. In contrast, the hand rehabilitation robot applies resistance to finger extension and grasping movements. This mode is characterized as resistance rehabilitation training.

### Outcomes

#### Primary outcome

##### fNIRS data acquisition

The study utilized a portable near-infrared brain imaging system (Brite MKIII, Artinis, Netherlands) to monitor participants’ hemodynamic changes during rest and rehabilitation tasks (16). Data were synchronously collected and analyzed using the OxySoft software. The device setup included 10 transmitting and eight receiving optodes, arranged in a 2 × 12 channel layout to mirror the distribution characteristics of the brain’s hand motion area, accounting for the arrangement of regions of interest (ROI) from previous studies. This configuration resulted in 24 collection channels symmetrically distributed across the participants’ left and right hemispheres. The distance between the detectors and the light sources was set to 30 mm, emitting wavelengths of 763 nm and 842 nm at a sampling rate of 25 Hz. Participants rested and relaxed for 2 min, followed by a minute of rest with their eyes closed (resting state). Subsequently, they engaged in a 10-min rehabilitation exercise while near-infrared and surface electromyography signals were collected. The task and data collection concluded simultaneously, after which participants were asked to remain still for an additional minute. Event labels such as “A” and “B” denoted the data captured between the start and end of a task. Events A and B signified the commencement and conclusion of synchronized tasks for fNIRS-sEMG, respectively, including the rehabilitation tasks involving the use of a robotic assistive hand glove, which were manually synchronized by the researcher for activation and deactivation.

##### sEMG data acquisition

**Figure 3:**
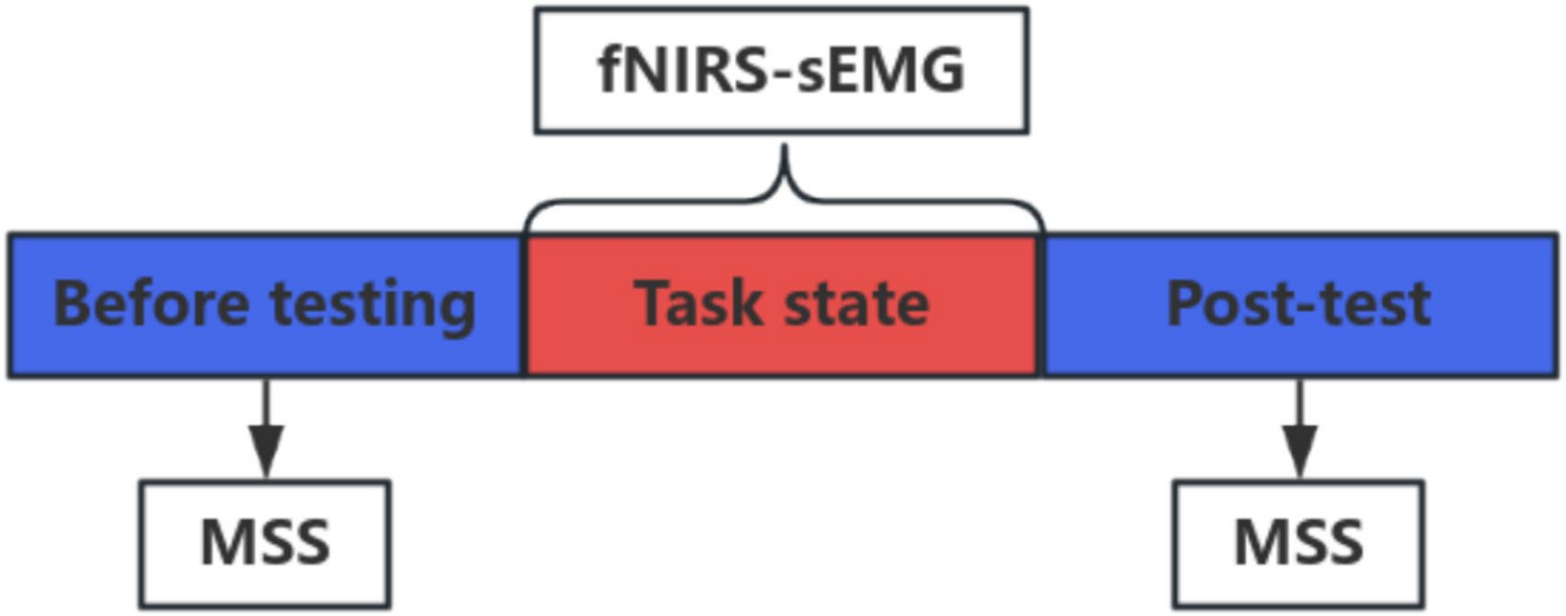
Test flow chart.

The BTS FREEEMG 300 wireless surface electromyography system (BTS Bioengineering, Milan, Italy) was utilized following the Surface Electromyography for the Non-Invasive Assessment of Muscles guidelines (17). This system, renowned for its wireless transmission technology, portability, and features such as high accuracy, long-distance telemetry, and durability is a reliable tool for surface electromyography acquisition and analysis in clinical diagnosis, rehabilitation assessment, and various research applications. The participants’ forearm hair was shaved, and the skin was cleansed with an alcohol swab to minimize skin resistance to the electromyography signal. Wireless surface electrodes were placed on the muscle bellies of the flexor carpi radialis (FCR), flexor digitorum superficialis (FDS), flexor carpi ulnaris (FCU), and abductor pollicis longus (APL) muscles. These electrodes were secured with medical tape and connected wirelessly to the computer, operating at a sampling frequency of 1,000 Hz. The test process is shown in Figure 3.

#### Secondary outcome

##### Function

The Motor Status Scale (MSS) test is a crucial assessment tool for evaluating hand function impairment in patients with stroke (18), focusing on hand function and motor control. The MSS scale was divided into shoulder, elbow, wrist, hand and other parts, in which the shoulder and elbow and forearm were graded at 6 levels (0, 1−, 1, 1+, 2−, 2), ranging from involuntary movement to normal movement, and the wrist and hand were graded at 3 levels (0, 1, 2). The shoulder and elbow forearm movement measurement includes 12 shoulder movements and 5 elbow and forearm movements, and also tests the patient’s ability to hold the last position (for 3–5 s) with 5 shoulder movements and 1 elbow movement, with a total score of 40. The wrist and hand measured 3 wrist movements, 15 hand movements, and 3 functional tasks of the hand, with a total score of 42. Higher scores, approaching the total score of 82, indicate better motor function.

### Data management

#### fNIRS data management

The OxySoft software package is designed to process the activation of oxygen concentration during task processes and generate 3D functional cortical activation maps of the brain. Initially, fNIRS data preprocessing is conducted using MATLAB software. The “oxysoft2matlab” plugin from Artinis company converts raw data files (*.oxy5 and *.oxyproj) into the *.nirs format. Subsequently, the Homer2 plugin transforms intensity data into Oxy-Hb. Motion artifacts across all channels are rectified, and any defective channels are identified. A bandpass filter is employed to eliminate noise generated by heartbeat, respiration, and low-frequency signal drifts, with a low frequency of 0.01 Hz and a high frequency of 0.2 Hz. The phase information of the 0.01–0.08 Hz signal is identified using wavelet transform methods. Changes in cortical frequency specificity are assessed by calculating wavelet amplitude, transverse index, and wavelet phase coherence (19). Due to its higher signal-to-noise ratio, Oxy-Hb (20) is selected as the indicator for analyzing oxygen concentration in subsequent analyses. The concentration of Oxy-Hb in relevant brain regions during task processes is calculated, with regions of interest (ROIs) including the prefrontal cortex (PFC), superior frontal cortex (SFC), supplementary motor area (SMA), and premotor cortex (PMC). The change in oxyhemoglobin for each channel is calculated by averaging the oxyhemoglobin across all tasks between events A and B. The average oxyhemoglobin concentration during three rehabilitation tasks with robotic-assisted hand function therapy is obtained. By dividing the sum of oxyhemoglobin concentrations in all channels within each ROI by the number of channels in the ROI, the average oxyhemoglobin concentration for each ROI during task states is determined.

#### sEMG data management

The acquired raw data undergoes bandpass filtering with a bandwidth of 20–500 Hz. Subsequently, the collected signals are analyzed using the EMGAnalyzer software to calculate the root mean square (RMS) value within the task interval (21). These assessments were conducted by two trained professional therapists, and the entire process was recorded. Subsequently, the average scores from two additional trained professional therapists were analyzed.

### Statistics

Statistical analysis was conducted using the SPSS software (Version 26.0, Chicago, IL, United States). Pearson correlation analysis assessed the relationship between the RMS and Oxy-Hb concentrations, and between different rehabilitation tasks and Oxy-Hb concentrations. Multivariate analysis of variance was used to examine whether there were differences in oxygenated hemoglobin concentration and RMS among the three rehabilitation tasks. Multiple postmortem comparisons were performed using LSD testing to identify differences between interventions and brain regions. Wilcoxon was used for data that did not conform to normal distribution. Descriptive statistics were reported as mean ± standard deviation (SD). The significance level for all statistical tests was set at 0.05.

## Results

### Accounting for all patients

The study enrolled 10 stroke patients with hand dysfunction. Finally, all 10 patients completed three robot-assisted hand function tests over a 5-day trial period, and the results were analyzed. No adverse events were reported during the study.

### Demographic characteristics

Ten participants completed the trial without any dropouts. Their mean age was 52.4 ± 6.3 years. There were no significant differences in age, gender, and baseline hand function among the three groups (see Table 1).

**Table 1:** Participant baseline characteristics.

Characteristic	Group A (*n* = 10)	*p*-value
Gender, male	4 (0.4)	0.731
Injury side, right	5 (0.5)	0.870
Onset period (W)	2.83 ± 2.82	0.817
Age (y)	62.67 ± 4.247	0.275
Height (cm)	162.51 ± 6.63	0.124
Weight (kg)	73.50 ± 9.11	0.544
BMI (kg/m^2^)	27.89 ± 3.62	0.116
MSS	41.8 ± 23.4	0.988

### Hand function

Following three sessions of robot-assisted hand function therapy, the patient’s MSS scores showed improvement (*p* < 0.05). Five days later, the baseline MSS score increased from 41.8 ± 23.4 to 46.7 ± 22.4 (see Table 2).

**Table 2:** Motor Status Scale scores for robot-assisted hand function therapy.

Test	Baseline	Post	*p*-value
MSS	41.8 ± 23.4	46.7 ± 22.4	0.001^a^

a *p < 0.05 comparing the baseline values against those after 5 days.*

### Brain function

**Figure 4:**
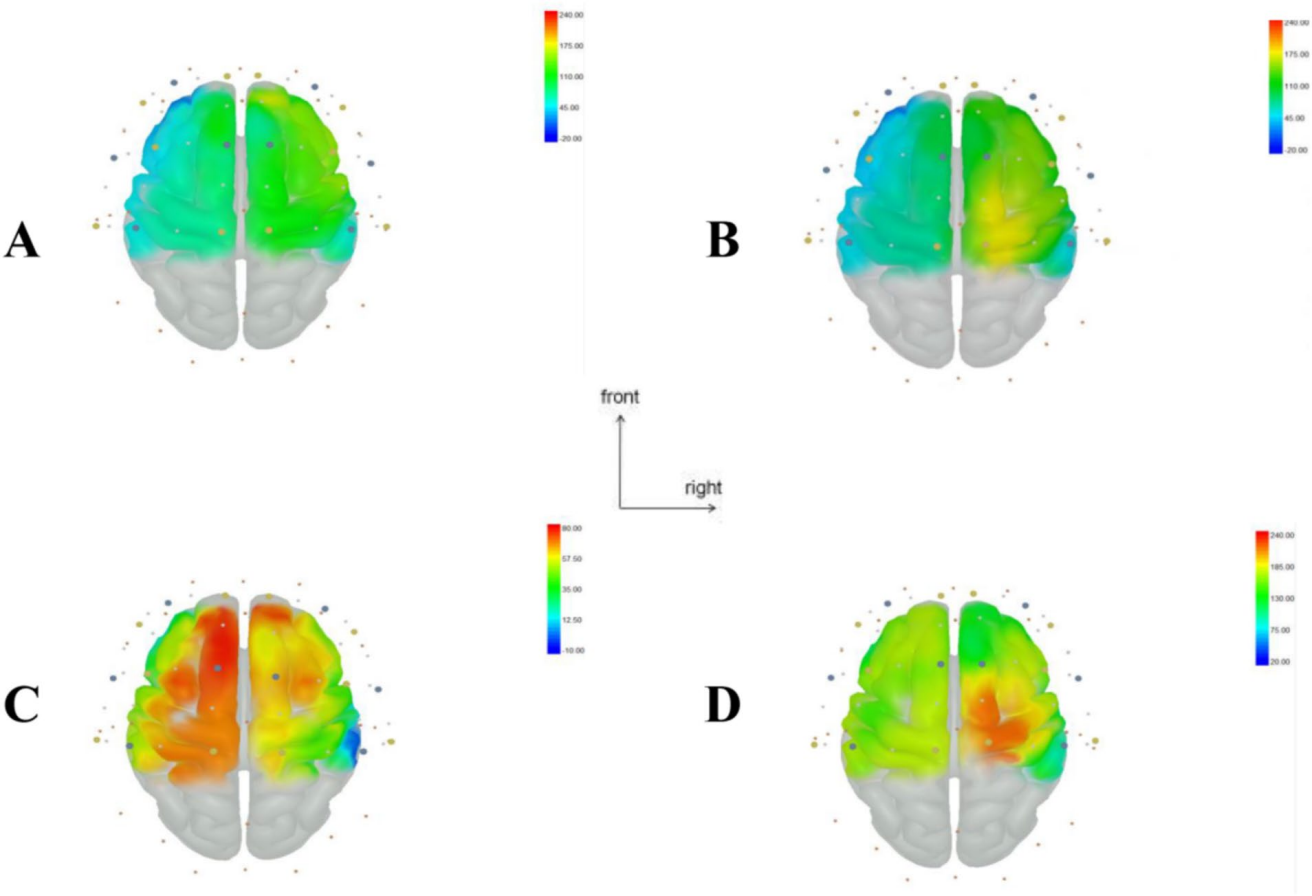
Visualization of regions of interest (ROI) in the brain during resting and task states in Groups A, B, and C. (A) Resting state brain functional area map. (B) Brain functional area activation map during passive rehabilitation task. (C) Brain functional area activation map during mirror task. (D) Brain functional area activation map during resistance task. This figure illustrates the change in oxyhemoglobin values, with larger *T* values, closer to red, indicating stronger activation in brain regions, while smaller *T* values closer to blue suggest weaker activation.

**Figure 5:**
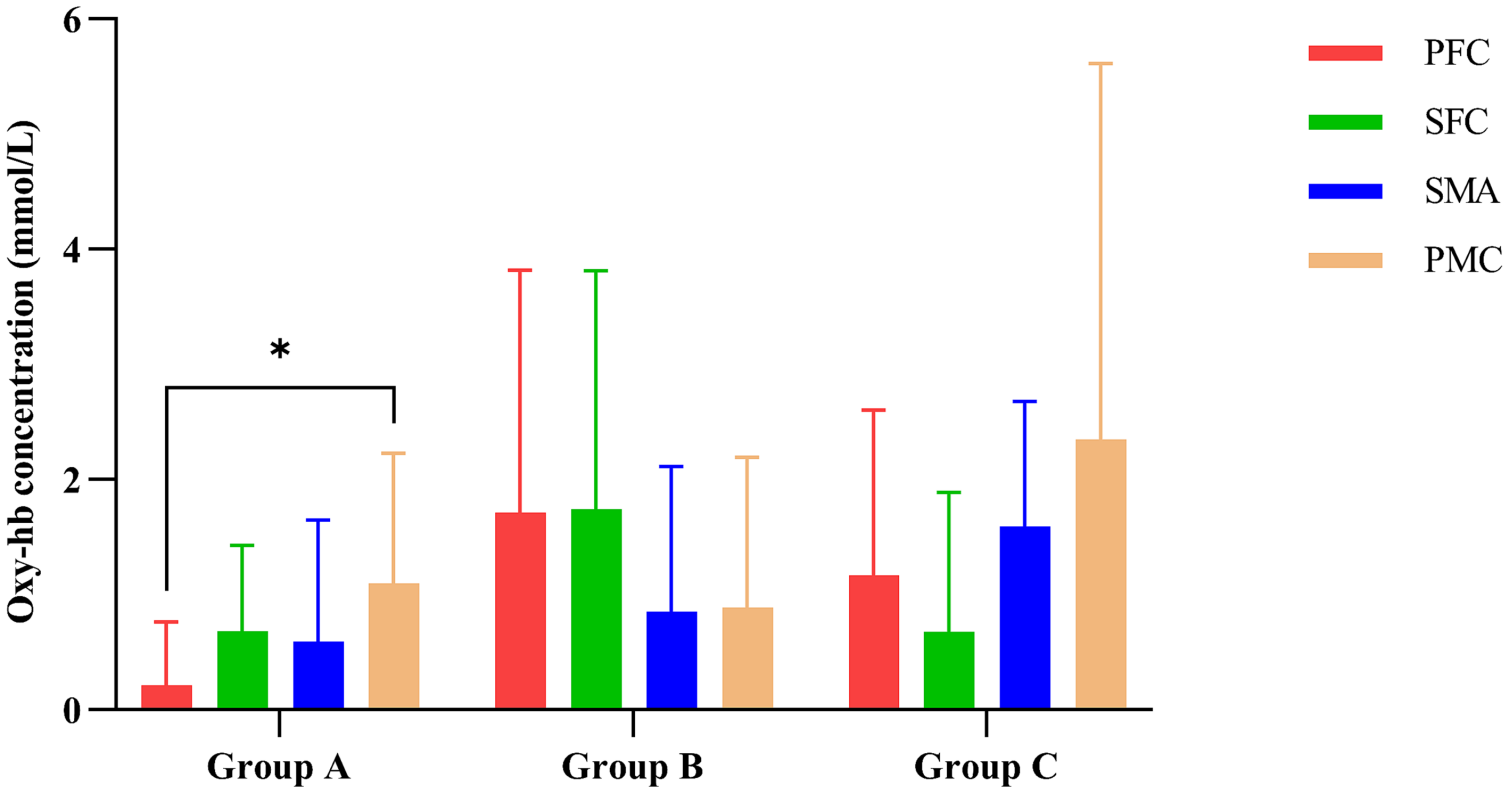
Impact of various rehabilitation tasks on the changes in oxygenated hemoglobin in the brain’s functional cortex. ^*^p < 0.05 indicates significant differences in Group A between the PFC and PMC.

**Figure 6:**
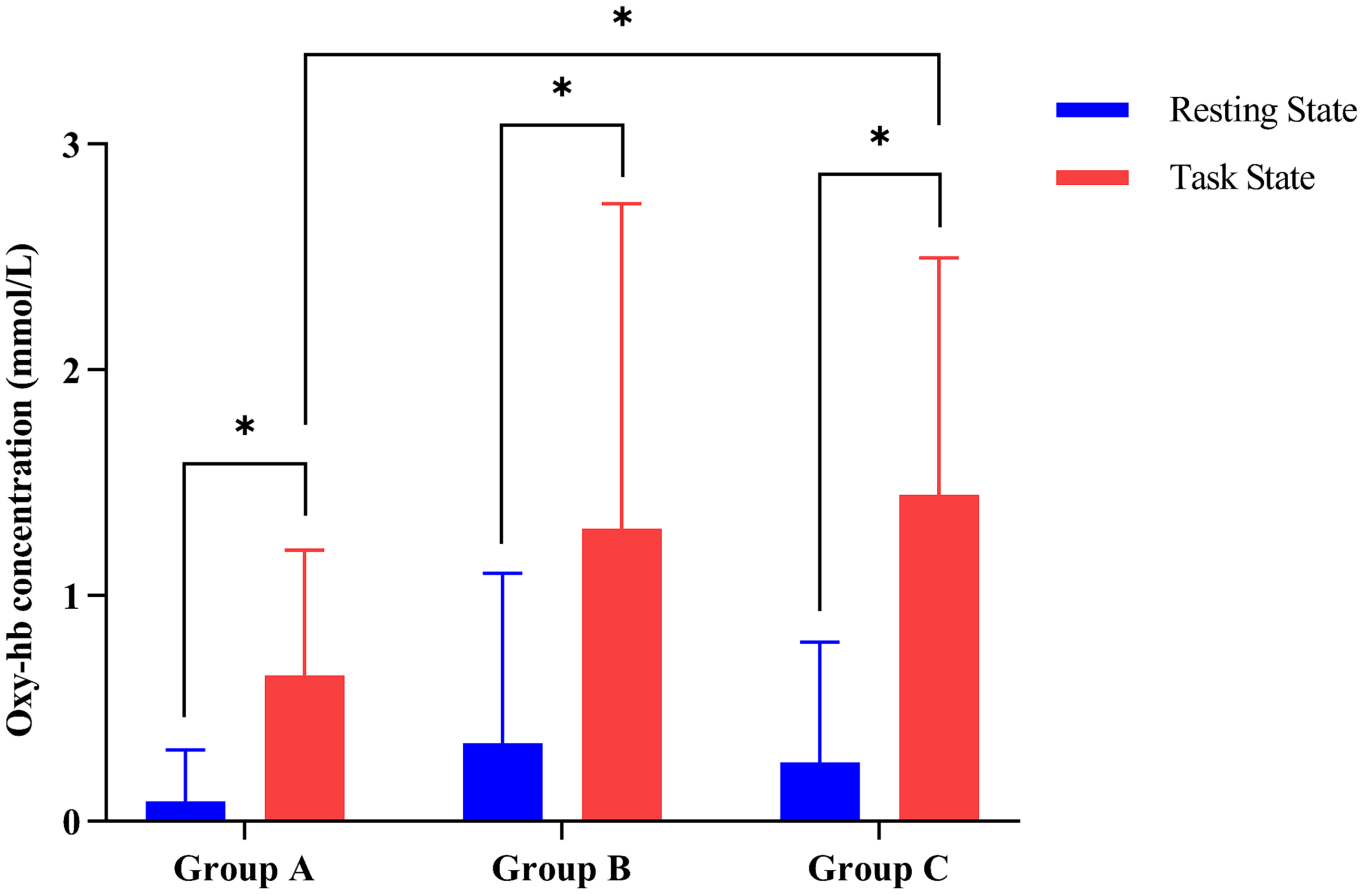
Contrasting changes in oxyhemoglobin levels between task and resting states in the three groups of robot-assisted hand function therapies. (Group A) Passive rehabilitation task. (Group B) Mirror rehabilitation task. (Group C) Resistance rehabilitation task. ^*^p < 0.05 indicated that there was statistically significant difference between task state and resting state oxygenated hemoglobin in the three groups.

As shown in Table 3, multivariate ANOVA showed differences in Oxy-Hb concentrations in the functional cortex of the brain for the three robot-assisted rehabilitation tasks, and between-group comparisons found significant differences in Hb concentrations between Groups A and B. Figure 4 shows significant changes in oxygenated hemoglobin concentration in brain function, with the cerebral cortex in different robot-assisted rehabilitation tasks where closer to red color indicates higher levels of activation. A showed that the activation of each brain region in the resting state was not significant. B showed that the activation level of PMC region in passive treatment task was higher than that in other brain regions. C showed that in the mirror therapy task, brain activation in the healthy hand was higher than that in the affected hand, but PFC and SFC on the affected side were also higher than that in other tasks. Compared to other brain regions, area D showed high levels of PMC and SMA activation during resistance therapy. Figure 5 illustrates that passive processing tasks have a small level of activation of brain regions, but significantly activate M1 and PMC. The mirror therapy task increased the activation levels of PFC and SFC, while the resistance therapy task enhanced the activation levels of SMA and PMC. Figure 6 demonstrates significant differences in Oxy-Hb concentration in the brain’s functional cortex between resting and task states across the three types of robot-assisted rehabilitation tasks (*p* < 0.05), brain regions in the task state were more activated than those in the resting state. The activation of brain function was more effective in the group with robot-assisted hand function therapy than in the group without robot-assisted hand function therapy.

**Table 3:** Comparative analysis of the impact of various rehabilitation tasks on changes in oxyhemoglobin levels in the functional brain cortex.

Test	Group A^b^	Group B	Group C^b^
PFC	0.213 ± 0.552^a^	1.710 ± 2.105	1.166 ± 1.438
SFC	0.681 ± 0.747	1.740 ± 2.070	0.680 ± 1.208
SMA	0.593 ± 1.056	0.851 ± 1.263	1.594 ± 1.082
PMC	1.100 ± 1.126^a^	0.886 ± 1.307	2.349 ± 3.265

a *p < 0.05 indicates significant differences in Group A between the PFC and PMC.*

b *p < 0.05 indicates a significant difference between Group A and Group C.*

### Muscle activity

**Figure 7:**
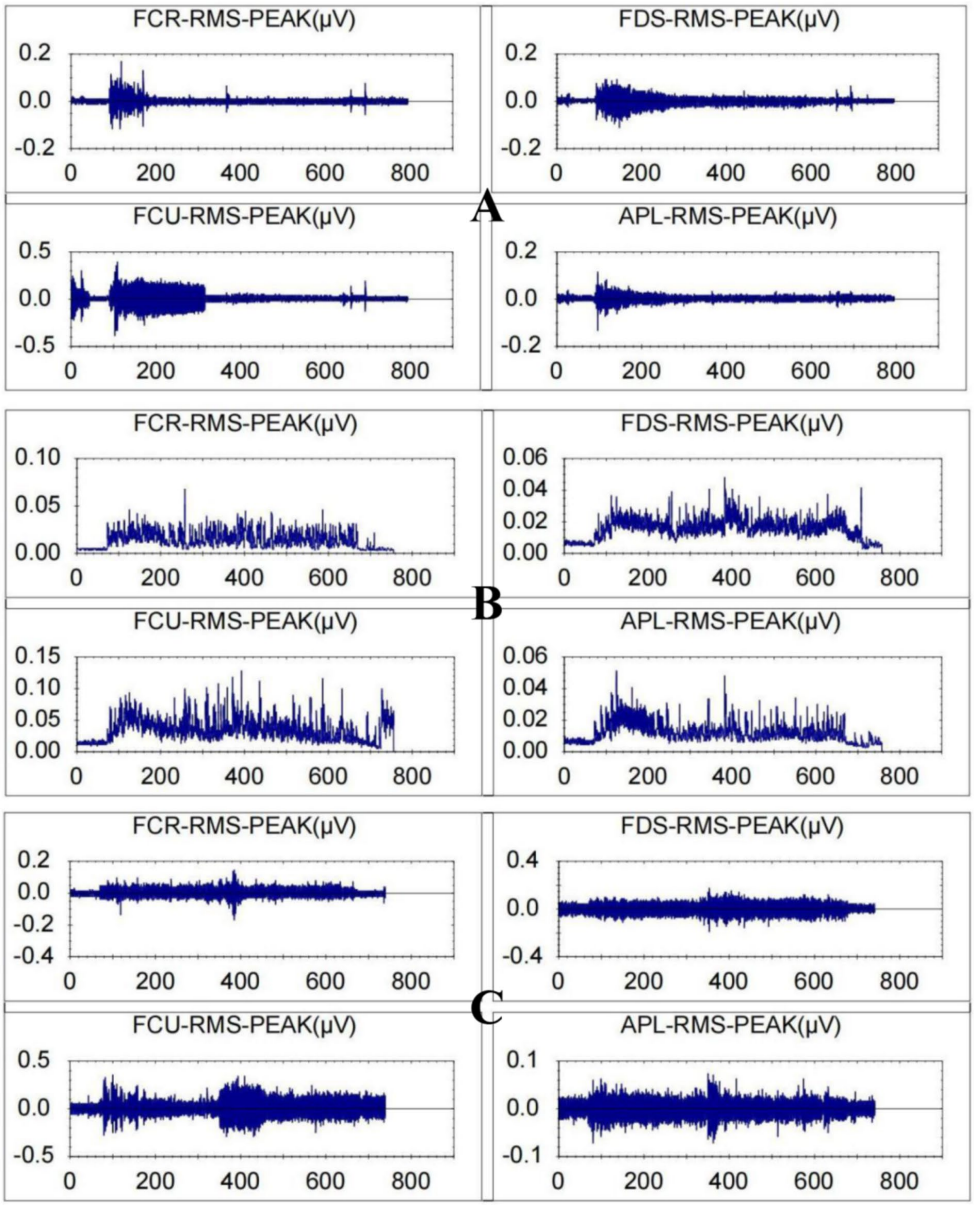
Root mean square values for a patient with stroke-related hand dysfunction during robot-assisted hand function therapy. (A) Passive rehabilitation task. (B) Mirror rehabilitation task. (C) Resistance rehabilitation task; RMS, root mean square; FCR, flexor carpi radialis; FDS, flexor digitorum superficialis, FCU, flexor carpi ulnaris; APL, abductor pollicis longus. The ordinate is in microvolts, and the abscissa is in seconds.

**Figure 8:**
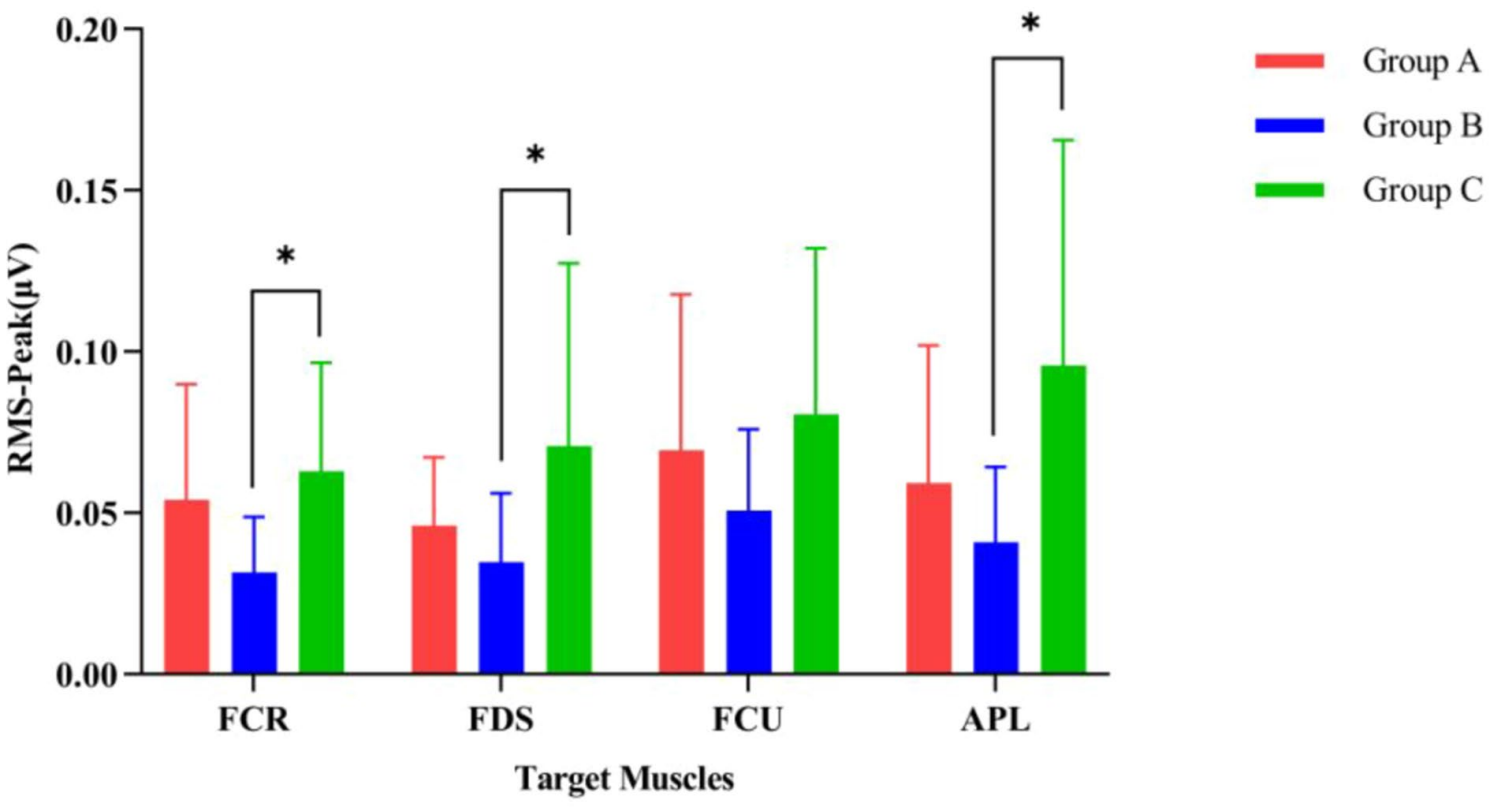
Comparison of the activation levels of arm muscles during three different rehabilitation tasks assisted by robotic gloves. (Group A) Passive rehabilitation task. (Group B) Mirror rehabilitation task. (Group C) Resistance rehabilitation task. RMS, root mean square; FCR, flexor carpi radialis; FDS, flexor digitorum superficialis; FCU, flexor carpi ulnaris; APL, abductor pollicis longus. ^*^p < 0.05 indicates that the arm muscles of the three groups are significantly different under exercise.

Figure 7 displays the magnitude of change in the RMS of the target arm muscles during rehabilitation tasks assisted by three types of robotic hands. Figure 8 compares the difference in RMS values of arm muscles across the three tasks. Analysis of variance showed that there was no significant difference in RMS of arm muscles among the three rehabilitation tasks (*p* > 0.05). Multiple comparisons showed that the intensity of target muscle activity in the resistance rehabilitation group was significantly higher than that in the mirror rehabilitation group (*p* < 0.05). There was no significant difference between the groups.

### Correlation analysis

**Figure 9:**
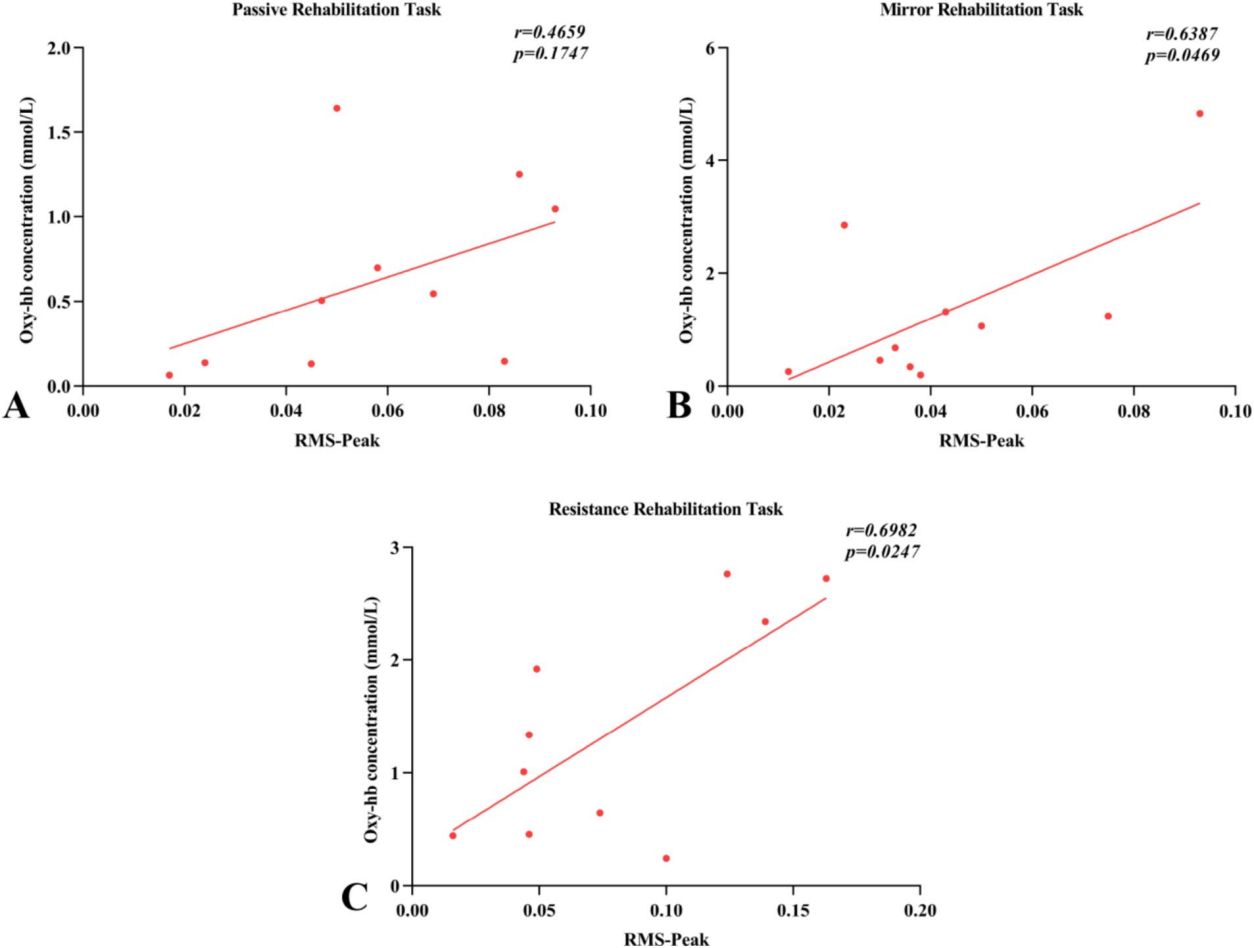
Pearson’s correlations between surface electromyography root mean square (RMS) and regions of interest (ROI) of fNIRS (oxyhemoglobin concentration) for the three rehabilitation tasks. (A–C) Indicated that the changes of blood flow movement in brain functional areas of passive rehabilitation task, mirror rehabilitation task and resistance rehabilitation task were correlated with Pearson of arm EMG-RMS.

Pearson correlation analysis revealed the relationship between the activation levels of arm muscles (RMS-peak) and the blood flow activity in brain regions (Oxy-Hb concentration) across three different rehabilitation task conditions involving robot-assisted hand function therapy (Figure 9). In passive rehabilitation tasks, there was a positive correlation between arm muscle activation (RMS) and Oxy-Hb data of cerebral functional cortex ROI, with a correlation coefficient of 0.4659, but the difference was not significant (*p* > 0.05). In mirror rehabilitation tasks, RMS displayed a significant positive correlation with the Oxy-Hb data of the brain functional cortex ROI, indicating a correlation coefficient of 0.6387 (*p* < 0.05). Similarly, in resistance rehabilitation tasks, RMS exhibited a significant positive correlation with the Oxy-Hb data of the brain functional cortex ROI, with a correlation coefficient of 0.6982 (*p* > 0.05).

## Discussion

### Innovation

#### Multimodal data fusion

This study combines fNIRS and sEMG for synchronous monitoring, offering a more comprehensive understanding of motor control and brain function. Unlike traditional research relying on single measurements, this enhances the assessment accuracy through the combination.

#### Focus on neuroplasticity research

This study provides a new perspective in stroke rehab by investigating neuroplastic changes in patients with robot-assisted therapy. The focus aims to clarify how it facilitates neuronal reorganization for motor function recovery.

#### Individualized rehabilitation for specific motor tasks

By capturing muscle and brain responses in various robot-assisted tasks, this research supports individualized programs. The tailored strategy meets diverse needs, enhancing rehab efficacy.

### Mechanisms of treatment

#### Motor learning and neuroplasticity

Compared with the resting state, the concentration of Oxy-Hb in the brain region of rehabilitation task is significantly increased, that is, the increase of cerebral blood flow, which is correlated with the increase of brain functional activity. When brain regions are active, their neurons consume more oxygen and require more efficient blood supply to meet oxygen and nutrient requirements. This complex relationship between brain activity and blood flow is called neurovascular coupling (22). Activation of functional brain regions suggests that robot-assisted therapy may promote neuroplasticity (23, 24). The results suggesting that robot-assisted hand therapy significantly increased cerebral blood flow to the region by activating a brain region associated with hand function, thereby promoting brain reorganization and functional recovery. Robot-assisted therapy triggers neural plasticity through standardized and repetitive motor training, stimulating motor neurons. This mechanism of improving neuroplasticity through motor learning and exercises is of great significance for rehabilitation after nerve injury.

#### Enhanced feedback mechanism

fNIRS and sEMG offer real-time feedback, enabling patients to understand their motor performance and brain activity. Robot-assisted therapy uses precise movement and feedback mechanisms to effectively guide patients to training, activating the motor cortex, sensory cortex (25), and other related neural pathways in the brain. This not only helps to improve the patient’s motor capacity, but may also further accelerate recovery by strengthening neural connections and promoting neuroplasticity. This helps them adjust exercise strategies promptly, improving learning and rehabilitation.

#### Bidirectional effects on the brain

Robot-assisted hand function boosts hand skills and activates brain regions for movement through specific tasks, promoting the neural basis of motion. Utilizing fNIRS to monitor blood flow changes (oxyhemoglobin concentration) in the brain, our findings indicate that various rehabilitation tasks assisted by robots exert distinct effects on the activation sites and levels of the brain cortex. Specifically, mirror therapy tasks notably enhanced the activation levels in the PFC and SFC, regions closely associated with higher cognitive functions, attention control, and motor planning (26). This association suggests mirror therapy can improve the brain’s attention and movement-planning capacity, stimulating these regions (27, 28). The resistance rehabilitation task significantly promoted the activation of SMA and PMC (29). The SMA is involved in the coordination of complex movements and the integration of sensory feedback (30), while the PMC plays an important role in the preparation and organization of movements (31). In resistance rehabilitation tasks, resistance training directly stimulates brain regions involved in movement execution and control by enhancing the muscle response to resistance, and increased activation of these regions suggests that resistance rehabilitation tasks help improve an individual’s motor skills and overall functional performance in strength training. Patients perform tasks not only physically but also through brain cognition, integrating movement and perception for better recovery.

#### Improved motor control and coordination

Robot-assisted training enhances motor control and coordination accuracy. Mirror rehabilitation tasks in post-stroke rehab help patients regain forearm and hand control, facilitating overall motor function recovery. While passive rehabilitation tasks had a minimal impact on the activation levels of targeted brain areas, they still exhibited some activation in the PMC region (32). This activation could be attributed to the brain’s processing and response to external movement stimuli during passive movements, particularly in regions implicated in movement planning and anticipation. Therefore, mirror and resistance rehabilitation tasks are more effective at activating brain areas associated with movement, a conclusion consistent with previous research (33).

### Relevance

The correlation between the RMS of sEMG, as measured by fNIRS, and the ROI signifies the synchronization or consistency observed in brain activity within a specific ROI with the electrical activity of muscles, as measured by EMG. This correlation reflects the relationship between muscle movement and brain activity.

#### Mechanism elucidation

Previous studies have demonstrated a significant association between muscle movement and brain activity, which can aid in comprehending the underlying causes of impaired hand function resulting from stroke.

During robotic assistance for hand function rehabilitation post-stroke, a correlation was observed between muscle movement activity in the brain’s cortical areas and Oxy-Hb concentration. Specifically, during muscle movement, the brain regions associated with planning and executing movements become more active (neuromuscular coupling) (34), necessitating increased oxygen and nutrients to support this neural activity. Consequently, blood flow to these areas increases (cerebral blood flow coupling) (35), resulting in Oxy-Hb concentration increase, indicating a close relationship between muscle movement and activity in specific brain regions. Mirror rehabilitation may enhance patients’ perception and imagination of movement (36), further activating brain areas related to movement planning and execution (37), such as the motor cortex, and frontal and parietal lobes (mirror training effect). This involvement in perception and imagination may activate muscle movement, particularly in passive, mirror, and resistance rehabilitation processes tailored to engage the brain’s movement-related areas (38). The sEMG signal, originating from the muscle electrical activity of the α-motor neurons in the spinal cord under brain motor cortex control (39), undergoes characteristic changes (amplitude and frequency) influenced by factors such as varying muscle activity levels and functional states. The cerebral cortex plays a pivotal role in muscle activity control, transmitting motor signals via nerve impulses, thereby eliciting muscle contractions (40, 41).

#### Clinical application

This comprehension provides a clinical foundation for designing more effective rehabilitation training methods aimed at assisting patients in regaining hand function. The correlation analysis between the fNIRS-sEMG synchronization method in the brain region and the Oxy-Hb concentration revealed that different rehabilitation tasks with robot-assisted hand functional therapy yielded varying effects on forearm muscle movement. Mirror rehabilitation promotes nerve regeneration and muscle coordination (42), resistance rehabilitation enhances muscle strength and endurance (43), and passive rehabilitation aids in muscle relaxation and improves joint mobility (44).

#### Enhancing effectiveness and speed

Training approaches that combine muscle movement and brain activity may lead to improved treatment outcomes and faster recovery by promoting neuroplasticity –the reestablishment and strengthening of neural connections in the brain. The resistance and passive rehabilitation groups demonstrated improved arm muscle strength in this study, while the mirror rehabilitation group exhibited comparatively modest gains. This observation is attributed to mirror rehabilitation’s incorporation of the healthy side hand to guide grasping movements, resulting in reduced muscle contraction compared to passive and resistance rehabilitation. Utilizing robotic assistance in hand function therapy prolongs arm muscle contractions, thereby improving muscle strength and functional recovery of the affected upper limb. This evidence collectively underscores the efficacy of robotic-assisted hand function therapy in treating post-stroke hand dysfunction (45).

## Limitations

This study has some limitations, such as small sample size and short study time. Future research directions include increasing the sample size, extending the study time, and exploring other modes of rehabilitation robot training.

## Conclusion

The present study revealed a significant correlation between muscle movement and brain activity after stroke, a finding that provides an important scientific basis for understanding the therapeutic mechanism of hand functional impairment. By combining fNIRS and surface electromyography (EMG), we were able to clarify the interaction between different movements and corresponding brain regions, which plays a key role in the recovery process of patients.

## Data Availability

The raw data supporting the conclusions of this article will be made available by the authors, without undue reservation.
